# Evolutionary implications of Liebig's law of the minimum: Selection under low concentrations of two nonsubstitutable nutrients

**DOI:** 10.1002/ece3.3096

**Published:** 2017-06-08

**Authors:** Omar M. Warsi, Daniel E. Dykhuizen

**Affiliations:** ^1^ Department of Ecology and Evolution Stony Brook University Stony Brook NY USA; ^2^ Department of Medical Biochemistry and Microbiology Uppsala University Uppsala Sweden

**Keywords:** *Escherichia coli*, experimental evolution, Liebig, magnesium limitation, nitrogen limitation

## Abstract

Interactions between different axes of an organism's niche determine the evolutionary trajectory of a population. An extreme case of these interactions is predicted from ecological theory in Liebig's law of the minimum. This law states that in environments where multiple nutrients are in relatively low concentrations, only one nutrient will affect the growth of the organism. This implies that the evolutionary response of the population would be dictated by the most growth‐limiting nutrient. Alternatively, it is possible that an initial adaptation to the most limiting nutrient results in other nutrients present in low concentration affecting the evolutionary dynamics of the population. To test these hypotheses, we conducted twelve evolution experiments in chemostats using *Escherichia coli* populations: four under nitrogen limitation, four under magnesium limitation, and four in which both nitrogen and magnesium are in low concentrations. In the last environment, only magnesium seems to limit growth (Low Nitrogen Magnesium Limited environment, LNML). We observe a decrease in nitrogen concentration in the LNML environment over the course of our evolution experiment indicating that nitrogen might become limiting in these environments. Genetic reconstruction results show that clones adapted to magnesium limitation have genes involved in nitrogen starvation, that is, *glnG* (nitrogen starvation transcriptional regulator) and *amtB* (transport protein) to be upregulated only in the LNML environment as compared to magnesium‐limiting environments. Together, our results highlights that in low‐nutrient environments, adaptation to the growth‐limiting nutrient results in other nutrients at low concentrations to play a role in the evolutionary dynamics of the population.

## 1 INTRODUCTION

Adaptation along different axes of an organism's niche has been the focus of many questions in evolutionary biology. It is inherently linked to niche evolution and to concepts of trade‐offs (Dykhuizen & Dean, 2004; Mole, 1994), evolutionary constraints (Futuyma, 2010; Mole, 1994), evolutionary rates (Bennett, Lenski, & Mittler, 1992; Bolnick, 2001), causal explanations of biodiversity (Fryer & Iles, 1972; Maharjan, Seeto, Notley‐McRobb, & Ferenci, 2006), stable community structures (Tilman, 2004), and outcomes of ecological competition (Futuyma & Moreno, 1988; Silvertown, 2004; Tilman, 2004). This evolutionary process has two aspects: adaptation along each axis of a niche and the interaction between different axes. The optimal evolutionary trajectory for a population is dependent on the nature of these interactions. Despite their importance, our understanding of how these interactions dictate evolutionary outcomes is very limited. In this study, we combine ecological theory and experimental evolution to predict and test the outcomes of these interactions. We use the niche axes of resource usage and Liebig's law of the minimum to analyze evolutionary outcomes for populations evolving in environments where the concentration of multiple nutrients is low, such that, if taken individually, each nutrient would limit growth.

Resource usage, as a selective pressure, has been extensively studied in the fields of both ecology and evolution (Dykhuizen, Dean, & Hartl, 1987 Sonti & Roth, 1989; Tilman, 2004). Previous experimental evolution studies investigating resource usage as a selective pressure have focused on cases where a single nutrient is in low concentration (Dykhuizen et al., 1987 Maharjan et al., 2006; Sonti & Roth, 1989; Wang et al., 2010), with only one study investigating the adaptive response using an experimental evolution approach under limitation of more than one nutrient (Walworth et al., 2016). The evolutionary response observed in the single nutrient‐limitation studies involves mutations that result in an increased efficiency of usage of the growth‐limiting nutrient, generally through an increased uptake of this nutrient (Dykhuizen et al., 1987 Maharjan et al., 2006; Sonti & Roth, 1989). Experimental evolution performed under conditions of co‐limitation of nutrients, specifically of iron and phosphorous (Walworth et al., 2016) displayed different adaptations in terms of growth rates and cell‐size changes between the co‐limited and single nutrient‐limited environments of iron or phosphorous.

Besides these two scenarios of nutrient‐limiting regimes (single nutrient limitation and co‐limitation by two nutrients), another selective pressure that can occur in an organism's natural habitat is when multiple nutrients are in low concentrations but only one nutrient dictates the growth of the organism. This directly follows from Liebig's law of the minimum that states that generally there is only one growth‐limiting nutrient (de Baar, 1994; Saito, Goepfert, & Ritt, 2008). To study how adaptation along the niche axis of resource usage for a single nutrient is either affected by or affects the niche axis of usage of another nutrient, we study the evolutionary response of a population in these environments where multiple nutrients are in low concentrations; however, only one is growth limiting. Although untested, a possible extrapolation of Liebig's law of the minimum is that in these environments, the evolutionary outcome is dependent only on the nutrient that results in growth limitation. In other words, the adaptive response of a population evolving in such environments will be dictated only by the growth‐limiting nutrient, and will mirror the adaptive response in populations evolving in single nutrient‐limiting environments; that is, from an evolutionary perspective there is no interaction between the axes of resource usage of the two nutrients. Alternatively, it is possible that as the population adapts to the most limiting nutrient, other nutrients that are present in low concentrations start affecting the evolutionary outcomes. Thus, even if the initial adaptive event is because of a single nutrient, the subsequent evolutionary changes could be a response to the low concentrations of both nutrients; that is, as the organism adapts to the niche axis of resource usage for a given nutrient, niche axes of resource usage of other nutrients start to influence the evolutionary outcomes.

In this study, we test these competing hypothesis using nitrogen limiting and magnesium‐limiting conditions, with the objective to gain insights into the adaptive responses in populations evolving in environments where both nitrogen and magnesium are in low concentrations. These two nutrients are unsubstitutable in terms of the physiological needs of the organism. We have conducted twelve evolution experiments in chemostats using *Escherichia coli*: four each under limiting nitrogen, limiting magnesium and where both are in low concentrations. We show that in the last type of environment that we choose, where both the nutrients are in low concentrations, limitation of magnesium limits population growth. We have thus named this environment as LNML (Low Nitrogen, Magnesium Limited).

We measured relative fitness of these evolving populations over 400 generations to show that the populations and clones adapt to each nutrient‐limiting environment. We also performed population sequencing at the end point of the evolution experiment to identify adaptive mutations under each nutrient‐limiting regime. We finally performed strain construction and gene expression analysis, which allowed us to specifically answer whether or not an initial adaptation to magnesium limitation in LNML environment results in low concentrations of nitrogen affecting cellular physiology, and by its extrapolation contribute to evolutionary dynamics.

## 2 MATERIAL AND METHODS

### 2.1 Strain and media used

The ancestor used in the study is a derivative of *E. coli* K‐12 MG1655. It is *lac‐* and *rpoS‐*. Davis minimal glucose media with different concentrations of salts was used for the long‐term evolution experiments. The media was made by adding 1.75 g potassium dibasic phosphate, 0.5 g potassium monobasic phosphate, 1 g ammonium sulfate, 0.5 g sodium citrate, and 0.1 g magnesium sulfate in one liter of water. Glucose was used at a concentration of 1 g/L. For the nitrogen limitation chemostat experiments, ammonium sulfate was used at a concentration of 0.05 g/L (0.7 mmol/L of nitrogen). Sodium sulfate was used to compensate for the sulfate concentration (0.9 g/L). For magnesium limitation chemostat experiments, no magnesium sulfate was added in the media. There was enough magnesium contaminating the other components of the media to give a limited growth. This media generally has excess of other trace elements, which allowed us to specifically limit these only for magnesium or nitrogen. In order to demonstrate nutrient limitation, population density at stationary phase was analyzed under various concentrations of ammonium ion and magnesium ions. For the long‐term evolution experiments, chemostats were changed every 10 days to avoid biofilm formation. The dilution rate in the chemostats was maintained at 0.33 hr^−1^ that results in a generation time of approximately 2 hr. Samples were taken every 24 hr and were frozen as glycerol stocks at −80°C. Contamination checks were performed every 24 hr by primarily screening the samples on minimal media plates containing 1% citrate as sole carbon source, where over the period of our evolution experiment no growth is expected. The chemostat experiments were allowed to run for 34 days, that is, 400 generations.

### 2.2 Identification of nutrient‐limiting environments

Twelve different nutrient combinations were tested to identify nutrient‐limiting regimes. These included four nitrogen concentrations (0, 0.07, 0.7, and 14 mmol/L) and three magnesium ion concentrations (0, 0.4, and 0.8 mmol/L). Nutrient limitation was predicted based both on population density at stationary phase and by performing the indophenol assay for the detection of the ammonium ion (Catalano, 1987). Cultures were allowed to grow in side‐arm flasks for 24 hr allowing each population to reach stationary phase. Cultures were removed from each flask, and spun down, and the supernatant was taken to detect presence or absence of nitrogen in the form of ammonium ions. Each environment was tested six times. In these experiments, we were only interested in whether or not nitrogen is present in the media, once the population had reached stationary phase.

### 2.3 Fitness measurements for populations and for end‐point clones

Fitness measurements for populations (at different time points) and for individual clones (isolated at the end point of the evolution experiment) were performed using competition assays. To measure fitness the *lac* operon (allowing usage of lactose as sole carbon source) was transduced into the ancestral strain by P1 transduction and was confirmed to be a neutral marker under conditions of nitrogen limitation, magnesium limitation, and in LNML environment by competing it with the *lac*‐ ancestor (Fig. S1). All competitions were carried out in chemostats under appropriate nutrient conditions. Each chemostat was inoculated with both the ancestral strain and the evolved population or evolved clone. The starting ratios in each case were 1:1. Competitions were carried out typically for 48–72 hr. Selection coefficient was calculated by plotting log of ratios of cell counts to time and calculating the slope of linearly regressed line. Relative fitness is calculated as (1 + selection coefficient), where the ancestor always has a fitness of 1. Each competition experiment was carried out twice. Error bars represent standard errors to the mean. t‐tests were used to detect statistically significant difference.

### 2.4 Next‐gen sequencing analysis

We extracted DNA using the DNeasy blood and tissue kit from Qiagen. Protocols were followed as mentioned in the manual, except the lysis time was increased to one hour. Pair‐end libraries were made using the Nextera XT sample preparation kit. Samples were dual‐indexed and pooled together. Illumina's Miseq was used for sequencing using the Miseq reagent kit v2 (500 cycle). Average coverage obtained for all the samples ranged from 7X to 22X. Geneious was used to map the reads onto the reference genome and to find mutations. For mutation detection, the cutoff values used were a minimum coverage of 10 and a minimum frequency of 40%. Only those mutations were called that were supported in both directions by setting a minimum strand bias *p*‐value of 10^−4^. From these identified mutations, potentially adaptive mutations were identified based on the following criteria: (1) A given gene consistently showing mutations among replicate experiments (Bull et al., 1997; Elard, Comes, & Humbert, 1996; Liao, McKenzie, & Hageman, 1986), (2) High‐frequency mutations that are unique to a population. Although we realize that these high‐frequency mutations can also be the outcome of hitchhiking or genetic drift, we think of these as adaptive responses because genetic drift is a weak force in populations as large as ours (~10^9^ cells). However, we cannot completely rule out neutral mutations reaching high frequencies hitchhiking with adaptive genetic changes. The population genomic data were also analyzed using Breseq (Deatherage & Barrick, 2014) to identify transposon movements or structural rearrangements that might have reached high frequency (40% or more) in the population or was present in replicate populations.

### 2.5 Mutant strain construction and measurements of selection coefficient

To measure the relative fitness of a given mutation in the three different nutrient‐limiting environments, strain construction was performed. A derivative of *E. coli* K‐12 MG1655 strain, which has been used previously for construction of the *E. coli* KEIO collection, was used as the starting strain for construction of the mutant used in this study (Datsenko & Wanner, 2000). Introduction of SNPs was performed using λ‐red recombineering (Datsenko & Wanner, 2000). Briefly, a *cat‐sacB‐yfp* cassette was PCR amplified from a plasmid using primers that consisted of 45 bp overhangs identical to sequence of the region where the SNP had to be inserted. This PCR product was then electroporated in a strain carrying the λ‐red plasmid, and the transformants were selected on chloramphenicol. The 45 bp overhang region allows the PCR product to be recombined in at the desired target position. Oligos containing desired mutation were then electroporated in these transformants, and negative selection on sucrose was performed. Confirmation of SNP was performed using Sanger sequencing. P1 transduction was used in the final step, where the *cat‐sacB‐yfp* cassette and the oligo, with the desired mutation, were introduced in a clean background. Using this method a double mutant Δ*yhaV phoQ* L467P was constructed. SNPs were confirmed using Sanger sequencing. Constructed mutants were also competed with the wild‐type allele using fluorescent markers. Briefly, yellow fluorescent protein and blue fluorescent protein were inserted in the *galK* gene in both the wild‐type strain and in the constructed mutants. These were then inoculated in equal proportions in chemostats with appropriate nutrient‐limiting conditions. The ratio of change of population densities was log transformed and plotted against time. The slope of this line was used as selection coefficient. Relative fitness is calculated as (1+selection coefficient), where the ancestor always has a fitness of 1. To remove the effects of fluorescent markers on growth rate in each environment, competitions were also performed by swapping the dyes between the wild‐type and mutant strain. Average of the selection coefficient is reported and error bars represent standard errors.

### 2.6 qPCR analysis for gene *glnG* and *amtB*

To gain insights into how a given nutrient‐limiting environment affects the cellular physiology, we performed qPCR analysis for genes that are overexpressed under nitrogen‐limiting conditions, that is, *glnG (*Nitrogen starvation transcriptional regulator) and *amtB* (ammonium ion transport protein) (Hua, Yang, Oshima, Mori, & Shimizu, 2004). For RNA extraction, cells were allowed to grow to stationary phase under appropriate nutrient‐limiting environments in 10‐ml culture volume. 5 ml of the culture was used for RNA extraction. RNA extraction was performed using the RNeasy Mini Kit (Qiagen) as per the manufacturer's protocol. Extracted RNA was DNase treated using the Turbo DNA‐free kit (Ambion) as per the manufacturer's protocol. The DNA‐free RNA was run on a 1% gel for visual inspection. 500 ng of RNA (quantified using the Qubit RNA BR assay kit) was used for cDNA preparation using the High Capacity Reverse Transcription Kit (Applied Biosystems). RT‐qPCRs were performed using the PerfecTa Sybr Green SuperMix (Quanta Biosciences). The housekeeping genes used as reference in the analysis were *cysG* and *hcaT*. The transcript abundance for *glnG* and *amtB* was normalized to the geometrical mean of the levels of *cysG* and *hcaT*. Three biological replicates and three technical replicates were used in each case. The averages and standard deviations shown in the figures are based on biological replicates.

## 3 RESULTS

### 3.1 Identification of nutrient‐limiting concentrations

**Figure 1:**
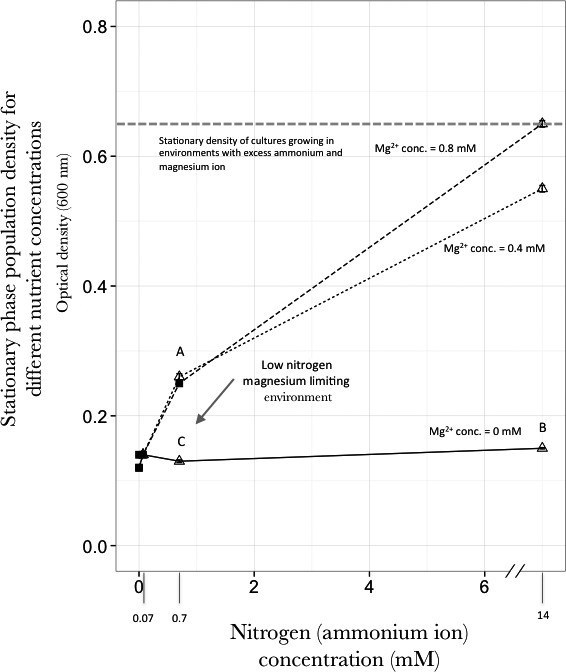
Identification of concentrations of nitrogen and magnesium that are growth limiting. The stationary phase population density of cultures was measured for twelve combinations of nitrogen and magnesium concentrations. The nitrogen concentrations used are 0, 0.07, 0.7, and 14 mmol/L. Each curve represents a given magnesium ion concentration (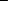 for 0 mmol/L, ‐ ‐ for 0.4 mmol/L and – – for 0.8 mmol/L). Nitrogen‐limiting concentrations represent conditions where ammonium was not detected using an indophenol assay (■). Δ represents environments where nitrogen was detected. Experimental evolution was performed at concentrations denoted by A (0.7 mmol/L nitrogen, 0.8 mmol/L magnesium), B (14 mmol/L nitrogen, 0 mmol/L magnesium), and C (0.7 mmol/L nitrogen, 0 mmol/L magnesium), which represents nitrogen‐limitation, magnesium‐limitation, and LNML environment, respectively

Before we ran the experimental populations of *E. coli* in chemostats, we needed to know the concentrations of nitrogen and magnesium that would make each of these the limiting nutrient. To do this, *E. coli* was grown in flasks for 24 hr with four different concentrations of nitrogen and three concentrations of magnesium, giving a total of twelve different conditions. The population density (Figure 1) and the presence/absence of nitrogen were measured (indophenol assay for ammonium ion concentration) for each environment at the stationary phase of growth. The lines in Figure 1 represent the different concentrations of magnesium. There was enough contaminating magnesium in the other components of the media that there was significant growth of the culture when no magnesium was added to the media. Thus, we used 0 mmol/L magnesium as one of the concentrations of magnesium tested. There was a little growth when no nitrogen was added to the media. Going from left to right in Figure 1 represents increasing nitrogen concentration. A positive slope of the line between two points implies nitrogen is limiting. If it is flat, it implies limiting magnesium.

In environments where a particular nutrient is limiting, the concentration of that nutrient should be negligible and in most cases undetectable. Using the same rationale, limitation by a different nutrient in an environment will leave the other nutrients at measurable concentrations. Six of twelve nutrient combinations showed the presence of ammonium ion (open triangles in Figure 1), and six nutrient combinations showed no ammonium ion present (closed square in Figure 1). Except for one point (0.7 mmol/L nitrogen, 0.4 mmol/L magnesium), the results for population density at stationary phase for different environments and the presence/absence of nitrogen fit nicely with each other. At 0 mmol/L magnesium, magnesium is the limiting nutrient except when nitrogen is 0.0 mmol/L. Nitrogen is limiting for the other two concentrations of magnesium, except when nitrogen is 14 mmol/L. At 14 mmol/L nitrogen concentrations, magnesium is limiting at 0.4 mmol/L, and something else (possibly oxygen) is limiting at 0.8 mmol/L (Figure 1). In environments where nitrogen concentration is 0.0 mmol/L or 0.07 mmol/L (for different concentrations of magnesium), the stationary phase population density is very similar to that observed in the environment with 0.0 mmol/L nitrogen and 0.0 mmol/L magnesium. This implies that the population growth in these environments is very limited and might even result in the eventual dying out of the whole population.

For the exceptional environment (0.4 mmol/L magnesium and 0.7 mmol/L nitrogen) where nitrogen was detected in the environment at stationary phase, the stationary phase population density show that nitrogen is the limiting nutrient. The concentration of nitrogen in this environment is low, as is suggested by the lesser intensity of blue color of the indophenol assay for nitrogen for this environment than all the others where there was excess nitrogen. However, this evidence for a low level of nitrogen in the environment was not an artifact of a particular experiment (contaminated tube, pipet tip, etc.). The experiment was repeated six times with different cultures, reagents, tips, and tubes, always with the same result. The population density in this environment is the same as in the environment with 0.8 mmol/L magnesium and 0.7 mmol/L nitrogen, showing that nitrogen is the limiting nutrient. We think that these observations might be the result of different cellular physiologies at the lower and higher concentrations of magnesium. At the lower concentration of magnesium, uptake of nitrogen is not as efficient as at the higher concentration. This suggests that the uptake and use of nitrogen and magnesium are not completely independent as proposed by Liebig's Law. This phenomenon would be interesting to study, but, for our evolution experiments, we choose environments where the nutrient limitation was better defined. Thus for the chemostat evolution experiments, we chose three environments: The one with limiting nitrogen is 0.7 mmol/L nitrogen and 0.8 mmol/L magnesium (A in Figure 1). The one with limiting magnesium is 0.0 mmol/L magnesium and 14 mmol/L nitrogen (B in Figure 1). The LNML environment (low nitrogen and magnesium limiting) is 0.0 mmol/L magnesium and 0.7 mmol/L nitrogen (C in Figure 1). For the LNML environment, the population density at stationary phase was identical to that expected under the single nutrient limitation for magnesium and lower than expected under nitrogen limitation (Figure 1).

### 3.2 qPCR measurements for *glnG* and *amtB* genes in the three selected environments

**Figure 2:**
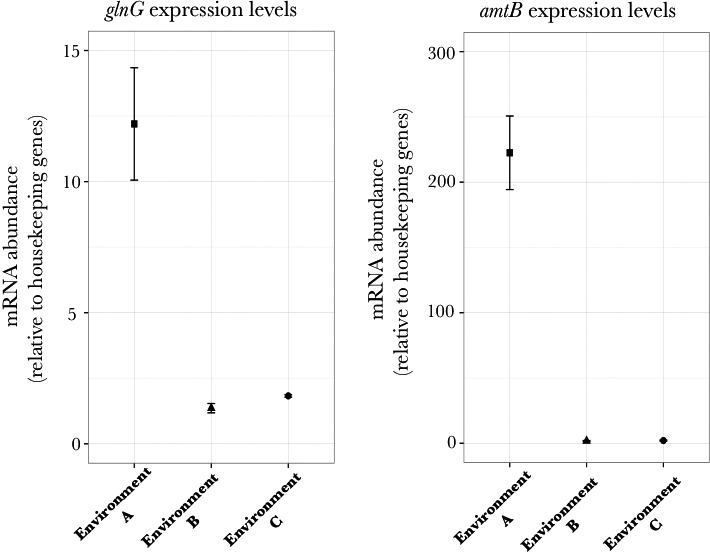
*glnG* and *amtB* gene expression levels in *Escherichia coli* MG1655 in environment A, B, and C. qPCR analysis shows statistically different expression levels for genes *glnG* and *amtB* as compared between environment A and environment B, as well as between environment A and environment C (*p *< .01). The difference between environment B and environment C is not statistically significant (*p *> .01). Samples were taken after populations reached stationary phase in the appropriate nutrient‐limiting environments

To further confirm the nature of nutrient limitation in the three environments chosen in the previous section, qPCR analysis was performed to measure the expression levels of genes *glnG* and *amtB,* once the populations had reached stationary phase in the three different environments. *glnG* codes for a protein that functions as a nitrogen response regulator and the *amtB* gene encodes for a protein that functions as an ammonium ion transporter, both of which are overexpressed under nitrogen‐limiting conditions (Hua et al., 2004). qPCR measurements show that in environment A, these genes are overexpressed as compared to both environments B and C (Figure 2). This difference is statistically different (*p *< .01) between environment A and environment B, as well as between environment A and environment C (*p *< .01). However, it is not statistically different between environment B and environment C (*p *> .01). This confirmed our results that in environment A the population growth was limited by low concentrations of nitrogen; and importantly in environment C the population growth of the cells was not limited by low concentrations of nitrogen.

### 3.3 Populations show complicated evolutionary dynamics under nutrient‐limiting conditions

**Figure 3:**
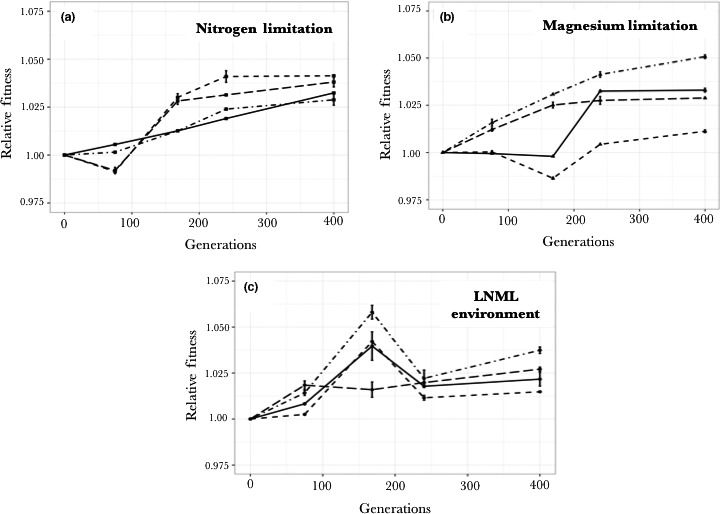
Fitness changes in 12 independent populations over four time points: 72, 168, 240, and 400 generations. Selection (Relative fitness −1) was measured by performing competition experiments against the ancestor in the same media in which the population evolved, regressing the ratios of population densities on time, and by measuring the slope. Error bars represent standard error. Different line types represent different replicates for each nutrient‐limiting environment

**Figure 4:**
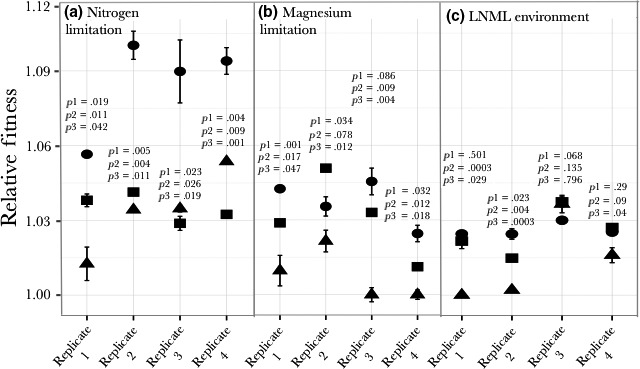
Fitness of clones compared to the fitness of the populations from which they were isolated. Evolved clones and evolved populations were competed against the ancestral population in the nutrient‐limiting conditions in which they evolved. *p*1 is the probability that the fitness of the clone and the population from which it evolved are the same. It is predicted that the fitness of the clone (●) and the population from which it evolved (■) will be the same (*p*1* *> .05), but in seven of twelve cases the fitness of the clone is significantly higher than the fitness of the population from which it came. The fitness of the clone was also measured under nutrient replete conditions (▲) to infer general adaptation that is not the result of nutrient limitation. It is expected that the fitness of the clone under nutrient replete conditions should be significantly less that the fitness of either this clone or the population from which it was isolated under appropriate nutrient‐limitation environment. *p*2 is the comparison between the fitness of the clone in a nutrient replete environment and the appropriate nutrient‐limited environment. *p*3 is the comparison between the fitness of the clone in a nutrient replete environment and the population in a nutrient‐limited environment. The expectation is met in ten of the twelve replicates. In one replicate (#3 on LNML), all three fitnesses (the clone, the population, and the clone in nutrient replete environment) are the same suggesting that all the fitness increase is unrelated to evolution to the limiting nutrient

While there was an increase in relative fitness for each of the evolving populations at 400 generations, replicate populations showed different trajectories of fitness increase (Figure 3). The fitness trajectories of populations evolved in the nitrogen‐limited chemostats show two patterns. Two replicates have a regular increase in fitness over the 400 generations that the experiments were run, ending at a relative fitness of 1.028 and 1.032 (Figures 3 and 4). The fitness trajectories of other two chemostats show an unusual pattern. The fitness at first decreases, then rapidly increases and plateaus, ending at relative fitness values of 1.038 and 1.041 (Figures 3 and 4). The fitness trajectories of the populations evolved under magnesium limitation were even more diverse. The fitness trajectories of two populations have a regular increase in fitness, ending at relative fitness values of 1.050 and 1.028 (Figures 3 and 4). The fitness trajectory for the third population has little increase until about 200 generation, then a jump in fitness followed by no further increase for the last 175 generation, ending at a relative fitness value of 1.033 (Figures 3 and 4). The fitness trajectory of the last population is negative at 175 generation, ending at a relative fitness of 1.011 after 400 generations (Figures 3 and 4). Populations evolving in LNML environment had relative fitness values ranging from 1.015 to 1.037 and showed a different pattern of fitness change as compared to populations evolving under single nutrient limitation. In three of these populations the fitness increased for the first 168 generations, only to then decrease over the next 100 generations, and then increase again (Figure 3c). The fourth population showed a rapid initial increase in fitness over the first 72 generations, and then little increase over the remaining 328 generation to end at a relative fitness of 1.027. Three of the populations evolving in the LNML environment show fluctuating changes in fitness suggesting complex selective dynamics for these populations.

### 3.4 Differences between relative fitness of clones and populations

To study if the clones from the evolved populations are representative of the populations themselves, we isolated clones from the populations at 400 generations and competed these clones against the ancestral clone in the same environment as the clones evolved. Of the twelve clones isolated, seven were significantly fitter than the population from which they were isolated (*p *< .05, Figure 4). For the remaining five, four were not significantly different in fitness than the population from which they were isolated and one was less fit (Figure 4). The fitness for most of these clones was statistically different between nutrient limiting and nutrient nonlimiting conditions (media with excess of nutrients present, Figure 4) demonstrating that most of the fitness increase observed in these clones can be attributed to nutrient‐specific adaptation. On the other hand, the fitness of one of the clones isolated from populations evolving in LNML environment (population 3) does not show any statistical difference when measured on nutrient limiting and nutrient nonlimiting conditions. The fitness of this clone is also the same as the fitness of the population from which it was isolated, suggesting that the increase in relative fitness of this population might be attributed mostly to background adaptation. We also observed cases where the relative fitness of the clone in the nutrient nonlimiting environment is equal to or greater than the fitness of the population in the nutrient‐limiting environment. Three of the four clones from the nitrogen‐limiting population show this phenomenon (Figure 4).

### 3.5 Clones adapted to LNML environment also show increased fitness under single nutrient‐limiting conditions

**Figure 5:**
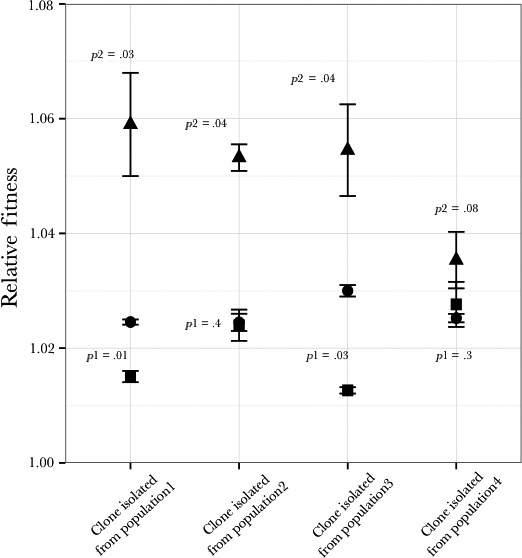
The adaptation of clones evolved in LNML to a limiting nitrogen environment (■) and a limiting magnesium environment (▲) compared with the adaptation in LNML(●). *p*1 is the probability that the adaptation to LNML (●) and to the nitrogen‐limiting environment (■) is the same. *p*2 is the probability that the adaptation to LNML(●) and to the magnesium‐limiting environment (▲) is the same

Relative fitness of clones adapted to LNML environment was also measured on single nutrient‐limiting conditions of nitrogen and magnesium. Each clone showed an increase in relative fitness under limiting magnesium conditions. Additionally, this increase in fitness in the magnesium‐limited environment was greater than that seen in the LNML environment (Figure 5 and Table 1). The relative fitness in the nitrogen‐limited environment was always significantly greater than one (relative fitness of ancestor). In two cases (clones from populations 1 and 3), the fitness increase in the nitrogen‐limited environment was significantly less that the fitness increase in the LNML environment. The other two of these values were not significantly different from the fitness in the LNML environment.

**Table 1:** Relative fitness of clones that evolved on LNML environment (for 400 generations) on single nutrient‐limiting environments. *p* values in each cell indicates if the difference between the relative fitness value and one (relative fitness of ancestor) is statistically significant

Clone analyzed	Relative fitness of clones in		
	LNML environment	Limiting nitrogen environment	Limiting magnesium environment
Clone isolated from Population 1	1.024 ± 0.0004 *p* value = .005	1.015 ± 0.0009 *p* value = .02	1.058 ± 0.009 *p* value = .05
Clone isolated from Population 2	1.024 ± 0.001 *p* value = .01	1.024 ± 0.002 *p* value = .03	1.053 ± 0.002 *p* value = .01
Clone isolated from Population 3	1.03 ± 0.001 *p* value = .01	1.012 ± 0.0005 *p* value = .013	1.054 ± 0.008 *p* value = .046
Clone isolated from Population 4	1.025 ± 0.0007 *p* value = .009	1.027 ± 0.004 *p* value = .04	1.035 ± 0.004 *p* value = .04

Overall these results show that clones adapted to LNML conditions demonstrate a strong response to magnesium limitation, suggesting adaption to magnesium limitation. However, our results also show that clones evolved in LNML environment show a greater increase in fitness on magnesium limitation than in the LNML environment, suggesting that low concentrations of nitrogen might influence the evolutionary trajectories of populations evolving under LNML conditions.

**Figure 6:**
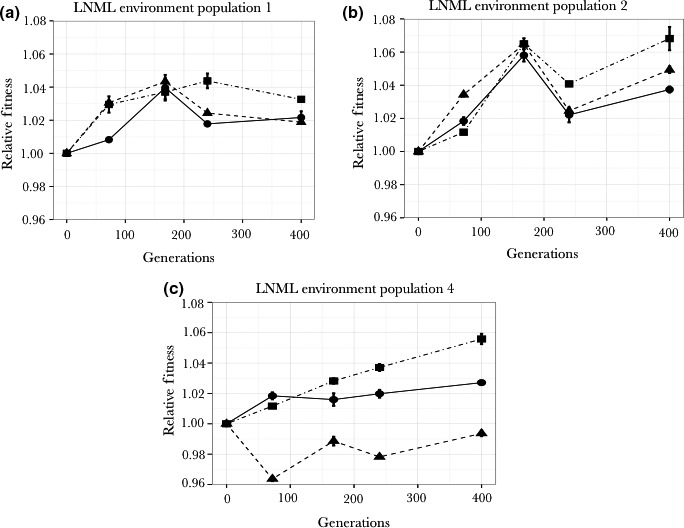
Fitness increase of three populations evolved in LNML over time in different environments in a LNML environment (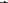), in a magnesium‐limited environment (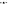) and in a nitrogen‐limited environment (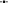). The samples were taken at 72, 168, 240, and 400 generations

To further elucidate the potential effect of low nitrogen concentrations, we measured the changes in fitness of populations adapted to LNML in limiting nitrogen and limiting magnesium environments (Figure 6). As the increase in relative fitness for population 3 is same under nutrient‐limiting and nutrient nonlimiting condition (Figure 4), we limited our analyzes to three populations (1, 2 and 4) that had evolved in LNML environment (Figure 6). Population 1 and Population 2 showed an overall increase in fitness in all three nutrient‐limiting environments. Population 4 showed an increase in fitness under magnesium‐limiting conditions and in LNML environment; however, it showed an initial decrease in fitness under nitrogen‐limiting conditions, only to increase back to a fitness of ancestral population. Importantly, each of these populations shows fluctuating fitness measures over time, highlighting the heterogeneity in the environment and in the evolving populations.

### 3.6 Identification of targets of selection using population sequencing

To study the nature of adaptive mutations in these environments, we performed population sequencing at the end point of the experiment for all our evolving populations. All the mutations that reached 40% or more are reported in Table S1. Here, we discuss only those genes that consisted of mutations present in replicate populations (i.e. to the same selective pressure) or that reached high frequency (40% or more) in a given population and are involved in magnesium or nitrogen metabolism. These criteria allowed us to understand the nature of the dominant clone/clones in these populations.

#### 3.6.1 Populations evolving under nitrogen limitation

We identified two potential targets of selection in populations evolving under limiting nitrogen conditions. These included nonsynonymous mutations in genes *glnG* and a 2 bp deletion in *glnL*. SNPs in *glnG* (NtrC) reach a high frequency (~52% and ~93%) in two of four of our populations, while the two bp deletion in *glnL* (NtrB) reaches fixation in the population in which it arose. Limiting nitrogen acts as the environmental signal for induction of gene *glnL* (protein NtrB), which activates the product of gene *glnG* (protein NtrC) by phosphorylating it (Gyaneshwar et al., 2005; Reitzer, 2003). NtrC induces the expression of multiple downstream operons that function to increase the efficiency of the cell under limiting nitrogen conditions and in scavenging for alternate nitrogen sources (Gyaneshwar et al., 2005; Reitzer, 2003). In our evolving populations, mutations in NtrB and NtrC occur in regions where the two proteins interact with one another. Previous studies on these proteins have shown that mutations in this region make NtrC insensitive to NtrB and result in constitutive (unregulated) expression of NtrC (Pioszak & Ninfa, 2004). None of these populations showed any transposon movement or structural rearrangement that was either repetitive between replicates or reached a frequency of 40% or higher in the population.

#### 3.6.2 Populations evolving under magnesium limitation

Two populations showed mutations in gene *yhaV*. One of this was a synonymous mutation 114A>G, while the other was a deletion of 11 bp in the gene. *yhaV* codes for the toxin in the toxin‐antitoxin system in *E. coli*. Other high‐frequency SNPs in these populations included nonsynonymous SNPs in genes *phoQ* (global regulator expressed under low‐magnesium concentrations) *and lptG* (involved in lipopolysaccharide transport for synthesis of outer membrane). None of these populations showed any transposon movement or structural rearrangement that was either repetitive between replicates or reached a frequency of 40% or higher in the population.

#### 3.6.3 Populations evolving in LNML environment

We found high‐frequency SNPs in genes *lptB* (cytoplasmic ATPase involved in lipopolysaccharide synthesis), *yhaV, rho* (involved in transcription termination), and *lptA* (involved in lipopolysaccharide transport for synthesis of outer membrane) across three replicates. It is interesting to note that one of the potential targets of selection under limiting magnesium conditions was gene *lptG*, which like *lptB* and *lptA*, is involved in synthesis of cell membrane. Also mutations in *yhaV* were seen for both LNML environment and magnesium‐limiting environment. We also found nonsynonymous mutation in the gene *adeD* to repeat between replicate populations. This gene codes for a deaminase and is involved in nitrogen metabolism. None of these populations showed any transposon movement or structural rearrangement that was either repetitive between replicates or reached a frequency of 40% or higher in the population.

### 3.7 Changing nutrient concentrations in LNML environment

**Figure 7:**
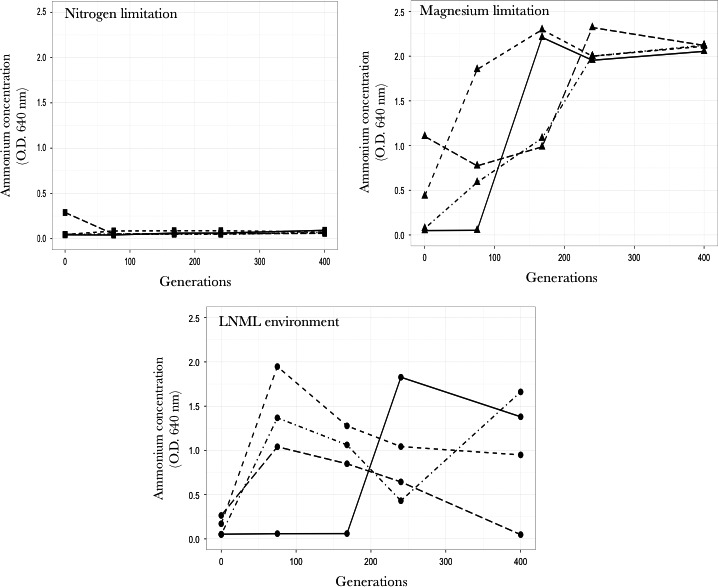
Changes in ammonium ion concentration during long‐term evolution experiments. Changes in concentration of ammonium ion under nitrogen‐limitation (■), magnesium‐limitation (▲), and LNML environment (●) over 400 generations. Under each limitation four replicate populations are represented as Population 1 (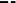), Population 2 (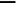), Population 3 (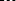), and Population 4 (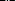)

To investigate the observed fluctuations in fitness in populations adapting to LNML environment, we measured ammonium ion concentrations at different time points in environments for each of the evolving population. To measure the ammonium ion concentrations, samples frozen directly from the chemostat were used to measure the change in ammonium ion concentration over the course of our experiments. All the populations start with lower ammonium concentrations than what is expected from the composition of the media. Presumably, a lot of ammonium is lost because of evaporation of ammonia from the media during the day and a half the chemostats ran before being inoculated with cells. In the nitrogen‐limited chemostats, the levels of ammonium ion was always below detectable levels (Figure 7). In the magnesium‐limited chemostats, while the pattern of ammonium ion increase differed between replicates, the increase in ammonium ion concentration was generally monotonic and at generation 400 reached similar high levels across all the four replicates (Figure 7). This increase suggests that more ammonium ion was being provided to this environment than could be utilized by the cells. In the LNML chemostats, three of the four populations show an initial increase in the concentration of nitrogen (ammonium ion). However after 72 generations, all of these show a decrease in ammonium ion concentration. This suggests that these populations are now using more ammonium ion than is being supplied in the chemostats, suggesting that these environments are moving toward becoming nitrogen limited. After this initial dip in ammonium ion concentration, three patterns are seen. In two LNML chemostats (those for population 2 and 4), the concentration of ammonium ion continues to decrease, while in population 3, the concentration of ammonium ion starts increasing (Figure 7). The fourth replicate (population 1) does not show any increase in ammonium ion concentration till 168 generation after which it shows an increase.

### 3.8 Low‐magnesium‐specific adaptive mutations have different relative fitness in single nutrient magnesium‐limiting environment and LNML environment

**Figure 8:**
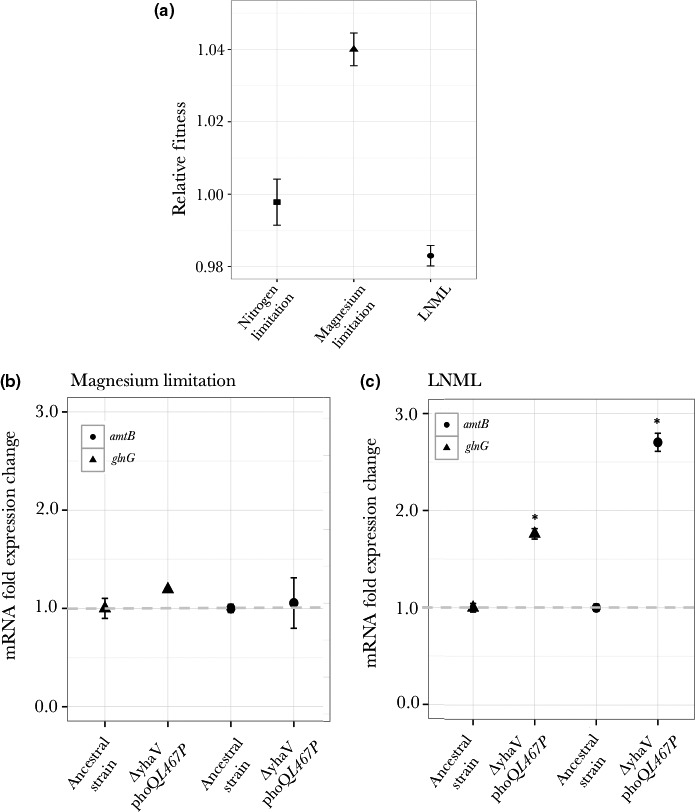
(a) Relative fitness measurements for a *ΔyhaV phoQ L467P* mutant under nitrogen‐limitation, magnesium‐limitation, and LNML environment. (b) Fold expression change for genes *glnG* and *amtB* under magnesium limitation for *ΔyhaV phoQ L467P* mutant relative to ancestral strain. (c) Fold expression change for genes *glnG* and *amtB in* LNML environment for *ΔyhaV phoQ L467P* mutant relative to ancestral strain. Asterisk indicates statistically significant difference (*p *< .01) between gene expression levels for the ancestral strain and mutant strain in a given environment

Given that our results show that over the course of the evolution experiment, ammonium ion concentration first increases in the LNML environment and then decreases, we hypothesized that this decrease might be an outcome of an initial adaptive event for magnesium limitation. If this were true, we would expect that a mutation that confers benefit under magnesium limitation would have a different effect on fitness in an LNML environment, as the low concentrations of nitrogen might now affect population dynamics. To test this hypothesis, we constructed a mutant consisting of two mutations: a deletion of gene *yhaV* and a *phoQ* L467P mutation (the combination of these mutations was identified to be beneficial under magnesium‐limiting conditions based on population genomic analysis of populations evolving under magnesium‐limiting conditions). When competed against a wild‐type strain under magnesium‐limiting conditions, this mutant has a fitness advantage of 4 percent (Figure 8a); while it has the same fitness as the wild‐type under nitrogen‐limiting conditions (Figure 8a). When competed against the wild‐type strain under LNML environment, we find that this mutant was in fact outcompeted, with the wild‐type having a fitness advantage of 1% over this mutant. These fitness measurements show that mutations that confer fitness advantage in magnesium‐limiting conditions, no longer do so in LNML environment.

### 3.9 Adaptation to low magnesium results in higher expression of genes involved in nitrogen uptake

Given that our results show that fitness advantage of a given mutation differed between magnesium‐limiting conditions and in the LNML environment (Figure 8a) and that ammonium ion concentrations decreases in the LNML environment (Figure 7), we hypothesized that this difference might be an outcome of the environment becoming nitrogen limiting once the magnesium‐specific mutation has become common in the population. In other words, once the population adapts to the most limiting nutrient, which is magnesium in our case, it faces limitation for the other nutrient, that is in low concentration, which is nitrogen in our experiments. To test this hypothesis, we measured expression levels of genes *glnG* and *amtB* for the *ΔyhaV phoQ* L467P mutant, which has a 4 percent fitness advantage over the ancestral strain under magnesium‐limiting conditions. Our results shows that compared with the wild‐type ancestral strain, this double mutant strain has a higher expression level for both these genes under LNML conditions (*p *< .01), while the expression levels are identical under magnesium‐limiting conditions (Figure 8b,c, *p *> .01). This indicates that a cell adapted to low‐magnesium concentration face nitrogen limitation in a LNML environment.

## 4 DISCUSSION

Liebig's law of the minimum states that in environments where multiple nutrients are in low concentrations, the most limiting of these will determine the population density of the organism. Figure 1 of this study shows that this law applies to bacteria. It is proposed that there will be selection to increase the efficiency of uptake and usage of the most limiting nutrient. If this is the case, Liebig's law can be extrapolated to predict evolutionary outcomes; for example, the array of genes selected should be the same for a limiting nutrient, irrespective of the concentration of other nutrients (the results from the magnesium‐limiting chemostats and from the LNML chemostats should be the same). The alternative hypothesis is that both nutrients have an affect on evolutionary outcomes: That is, Liebig's law does not give a complete picture of the evolutionary dynamics in environments where multiple nutrients are present in low concentrations. In our experiments, we find that populations evolving in environments with multiple nutrients in low concentrations are initially growth limited because of a single limiting nutrient (magnesium in our experimental setup). Consequently, these populations adapt to the limiting concentration of this nutrient. However, this initial adaptation results in other nutrients that are present in the environment at low concentration to start affecting, and possibly limiting, the population growth (nitrogen in our experimental setup). These varying nutrient‐limiting conditions results in changing selective pressures and selection intensities, leading to complicated evolutionary dynamics.

### 4.1 Adaptation in LNML environment is affected by low concentrations of both magnesium and nitrogen

As Figure 5 shows, LNML adapted clones are also clearly adapted to single nutrient magnesium limitation. However, there are two additional considerations regarding this observation. Firstly, the fitness increase of all LNML adapted clones is much higher (three of four at 5% significance) in magnesium‐limited chemostats than the fitness increase in LNML chemostats (Figure 5). Secondly, the fitness increase of LNML adapted clones in magnesium‐limited chemostats is much higher than the fitness increase for magnesium adapted clones in magnesium‐limited environments (Figures 4 and 5). The average fitness increase of the LNML adapted cultures in limited magnesium is 0.051 compared with 0.037 for the magnesium‐limited cultures in limited magnesium. This difference is not, however, statistically significant because of the very large range of fitnesses in each set (0.058, 0.053, 0.054, 0.035 for LNML and 0.043, 0.035, 0.045, 0.025 for magnesium limited). These results highlights two important points: 1. There is adaptation to magnesium limitation in the LNML adapted clones, 2. The adaptation to magnesium limitation is greater in the cultures adapted in the presence of low nitrogen levels than the cultures adapted in the presence of high nitrogen levels.

Our results suggest that an LNML environment is a complex environment, potentially consisting of temporally changing selective pressures. This fluctuating environment might result in this observed pattern of high adaptation to limiting magnesium conditions for the LNML adapted clones. Ketola et al. (2013) observed similar results in that populations adapting to fluctuating thermal environment had a higher increase in fitness at the mean temperature compared to the populations that were only adapted to this mean temperature. We do think that evolutionary dynamics in fluctuating environment can lead to the pattern we observe due to inefficient removal of deleterious mutations in populations evolving in a constant environment. Populations evolving under single nutrient‐limiting conditions, in most cases, show a monotonic increase in fitness. We have observed this (Figure 3) as have others (Wiser, Ribeck, & Lenski, 2013). This results in fitness epistasis for incoming mutations where the affect of both detrimental and beneficial mutations is reduced, especially when the population is close to a fitness maxima (Chou et al., 2011; Dykhuizen et al., 1987; Khan et al., 2011). Then, purifying selection is not being efficient, resulting in accumulation of mildly deleterious mutations. On the other hand, fitness trajectories of populations evolving in LNML environment show fluctuating patterns of selective pressure, which would make purifying selection more efficient.

### 4.2 LNML environment experience temporal changes in selective pressures: A changing niche space hypothesis

Our results show that populations evolving under environments of low‐nutrient concentrations show signatures of changing selective pressure. We supported this hypothesis by measuring the concentration of the nutrient that was initially nonlimiting but was present in low concentrations (nitrogen in our experiments). The concentration of nitrogen in the magnesium‐limited chemostats increases rapidly and reaches a high level at the end of the experiment. The concentration of ammonium entering the chemostat was 20‐fold lower in the LNML chemostats than the magnesium‐limited chemostats. Even yet the concentration of ammonium rose, as was expected since more nitrogen was being added to the chemostat than the cells could utilize. As the evolution experiment proceeded, the concentration of ammonium in the LNML chemostats fluctuates differently in different chemostats, sometimes increasing rapidly, sometimes dropping rapidly. The concentration of ammonia in chemostat 4 drops to zero at 400 generations, with a supposition that now nitrogen is the limiting nutrient in this chemostat. This could be because as Population 4 adapts to the limiting magnesium, it starts experiencing limitation to nitrogen.. The ammonium concentration in chemostats 2 and 3 rises quickly and then drops, suggesting that evolution for improved growth on limiting magnesium is causing a limitation in nitrogen. Our strain construction results confirm this hypothesis. A mutant, which is shown to be adapted to limiting magnesium concentrations, overexpresses genes involved in ammonium ion uptake in the LNML environment, but not in the magnesium‐limiting environment. This indicates that the lower concentrations of nitrogen are now affecting the population growth in the LNML environment and would thus play a role in the evolution of the population. Gorban et al. (2011), using theoretical models, demonstrated similar outcomes as a result of evolutionary adaptation: As the organism adapts to the demands of the limiting nutrient, other nutrients also become limiting. Our result also highlights another aspect of evolution of microbial populations under nutrient‐limiting conditions. We observe an unexpected pattern of decrease in fitness of populations across different time points, even when these populations are evolving under single nutrient limitations. We think that one potential reason for this unexpected result might be because these populations are “changing” the environments in which they are growing, perhaps by secretion of metabolites. Thus, the environment in which the populations evolve might be different from the environment where the population fitness was measured.

Our study also highlights a more general aspect of adaptation to multiple environmental factors. Different factors affect an organism's physiology in different ways; some might have a larger impact on the organism's physiology, while others might have a smaller impact. These factors define the niche space for the organism. Our results demonstrates that as an organism evolves to a given niche axis, the evolutionary potential along the other axes changes as well. In our experimental system, we start with two axes where only one determines the evolutionary dynamics of the population. This would imply that only this first axis is important in characterizing the niche space of the organism. However, adaptation along this first axis then increases the evolutionary potential along the second axis, increasing its role in defining the niche space of the organism. This idea of a changing niche space is slightly different from what is mostly found in literature, that is, either involving a spatial component or the concept of niche construction (Odling‐Smee, Laland, & Feldman, 1996; Pearman, Guisan, Broennimann, & Randin, 2008).

### 4.3 Cell‐membrane proteins and nitrogen metabolism proteins are potentially adaptive mutations in LNML environment

The potentially adaptive mutations in populations growing under LNML conditions include mutations in *yhaV, lptA, lptB, adeD,* and *rho*. *yhaV* was also found to be a target of selection under magnesium limitation. Also populations adapted to magnesium limitation show potentially adaptive mutations in gene *lptG*, while populations adapted in LNML environment show potentially adaptive genes in gene *lptA* and *lptB*. All three of these genes play a role in biosynthesis of cellular membrane. Thus, cellular membrane is a target of selection in both these environments. Magnesium ions play a role in stabilizing the cellular membrane, and it is possible that mutations in these genes result in the cellular membrane requiring less of magnesium ions for stabilization, allowing these to be used for other cellular processes. Characterization of the outer membrane, either through assays that check for surface hydrophobicity or for permeability, or a mass‐spec analysis of the outer membrane would help associate the genetic changes with the underlying physiological changes, and are currently being done. On the other hand, gene *adeD,* which function as a deaminase is assumed to be related to nitrogen limitation, selection for which might increase the efficient usage of nitrogen in the cell. Thus, we find that both low concentrations of magnesium and low concentrations of nitrogen affect these evolving populations. This correlates nicely with our observation of concentrations of ammonium varying in the LNML environment.

## 5 CONCLUSION

While this series of experiments was designed to explore evolutionary dynamics under different environmental conditions, it has important ecological considerations, that is, what differences in the environment gives significantly different evolutionary responses. Thus, experimental evolution can be used to define the environment from the organism's perspective rather than defined as scientists imagine it should be. In conclusion, our results highlight a concept of changing niche space as a result of natural selection, that is, adaptation along a given axes result in an increase of complexity of the niche. This changing niche space highlights why evolutionary dynamics in environments where multiple nutrients are in low concentrations is different from that when there is a single limiting nutrient. Our work supports Liebig's law for ecology (in the short term) but does not support an extension of his law to evolution and therefore to ecology over the long term, as it will be expected that evolution will tend to select for a phenotype where the growth is limited by an array of substances.

## CONFLICT OF INTEREST

The authors declare no conflict of interests.

## DATA ACCESSIBILITY

The fastq population sequences can be requested for to the corresponding author.

## AUTHOR CONTRIBUTIONS

D.E.D and O.M.W were involved in designing the experiments. O.M.W carried out the experiments, the genomic analysis and statistical analysis. D.E.D and O.M.W drafted and edited the manuscript.
