# Supplementary figures and images for: Evolutionary implications of Liebig's law of the minimum: Selection under low concentrations of two nonsubstitutable nutrients
Source: Ecol Evol. 2017 Jun 8;7(14):5296–309. doi: 10.1002/ece3.3096 (PMC5528229; doi:10.1002/ece3.3096)

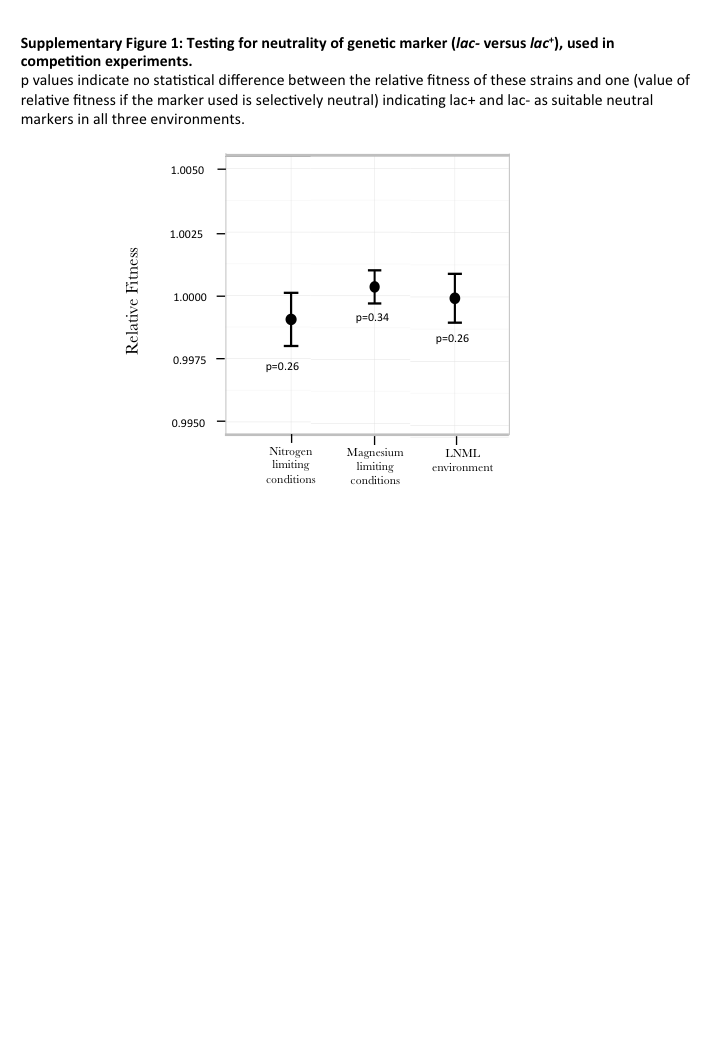

Supplement: Supplementary file 1 [file ECE3-7-5296-s001.tiff]
