# Supplementary material for: Evolutionary implications of Liebig's law of the minimum: Selection under low concentrations of two nonsubstitutable nutrients
Source: Ecol Evol. 2017 Jun 8;7(14):5296–309. doi: 10.1002/ece3.3096 (PMC5528229; doi:10.1002/ece3.3096)
Supplement: Supplementary file 2 [file ECE3-7-5296-s002.docx]

Supplementary table 1: List of non-synonymous mutations that reached 40% or higher in populations evolving under nitrogen limitation, magnesium limitation and in LNML environment.

| **Type of Nutrient limitation** | **Population/Replicate in which mutation is observed** | **Gene** | **Mutation** | **Physiological function** |
| --- | --- | --- | --- | --- |
| Nitrogen limitation  Environment | Population1 | *ycdT* | Phe157fs | diguanylate cyclase |
|  | Population1 | *gatC* | Val306fs | galactitol PTS permease |
|  | Population1 | *rph* | Ile225fs | RNase PH |
|  | Population 2 | *ydhB* | Ala45Gly | HTH (helix-turn-helix) transcription factor |
|  | Population 2 | *glnG* | Val18Leu | transcriptional dual regulator expressed under nitrogen limitation |
|  | Population 2 | *ygcQ* | His80fs | uncharacterized electron transfer pathway |
|  | Population 2 | *ruvB* | Glu293Gly | repair helicase |
|  | Population 2 | *gcd* | Asp414Asn | quinoprotein glucose dehydrogenase |
|  | Population 2 | *ldtC* | Leu180Pro | L,D-transpeptidase YcfS |
|  | Population 2 | *cysP* | Ser32Ty | sulfate / thiosulfate ABC transporter |
|  | Population3 | *ybdK* | Asp276Glu | carboxylate-amine ligase |
|  | Population3 | *focA* | Ala27Thr | formate transporter |
|  | Population3 | *rng* | Val108Gly | ribonuclease G |
|  | Population3 | *rtcA* | Ser217Phe | RNA 3'-terminal phosphate cyclase |
|  | Population3 | *mutM* | Val201Gly | formamidopyrimidine DNA glycosylase |
|  | Population3 | *glnL* | Gln11fs | NtrB sensory histidine kinase |
|  | Population3 | *hsrA* | Met18Val | uncharacterised member of the major facilitator superfamily (MFS) of transporters |
|  | Population3 | *ilvD* | Thr25Pro | dihydroxy acid dehydratase |
|  | Population3 | *zraR* | Val338Gly | ZraR transcriptional activator |
|  | Population3 | *yjiR* | Leu151Trp | predicted DNA-binding transcriptional regulator |
|  | Population3 | *yddE* | Phe25Ser | predicted diaminopimelate epimerase |
|  | Population3 | *ydhB* | Ala45Gly | predicted HTH (helix-turn-helix) transcription factor |
|  | Population3 | *ycjR* | Thr158Pro | predicted D-tagatose 3-epimerase |
|  | Population3 | *yeaG* | Phe265Leu | protein kinase |
|  | Population3 | *gltK* | Ala53Gly | glutamate / aspartate ABC transporter |
|  | Population3 | *uraA* | Ser317Phe | uracil:H^+^ symporter |
|  | Population3 | *yghQ* | Ala329Gly | predicted inner membrane protein |
|  | Population3 | *yidA* | Ile21Phe | sugar phosphatase |
|  | Population3 | *rtcA* | Ser215Gly | RNA 3'-terminal phosphate cyclase |
|  | Population3 | *rhsD* | His568Pro | RhsD protein in rhs element |
|  | Population3 | *gabP* | Trp100Gly | 4-aminobutyrate:H^+^ symporter |
|  | Population3 | *adeD* | Phe86Val | cryptic adenine deaminase |
|  | Population3 | *thiL* | Gln295Lys | thiamine monophosphate kinase |
|  | Population3 | *gmr* | Ser500Phe | cyclic di-GMP phosphodiesterase |
|  | Population3 | *frlA* | Leu54Val | fructoselysine / psicoselysine transporter |
|  | Population3 | *pnp* | Tyr380Asn | polynucleotide phosphorylase |
|  | Population3 | *nac* | Tyr148Phe | DNA-binding transcriptional dual regulator |
|  | Population3 | *yddW* | Ala355Glu | predicted lipoprotein |
|  | Population4 | *ttdB* | Gly60fs | L-tartrate dehydratase, β subunit |
|  | Population4 | *putP* | Ala337Thr | proline:Na^+^ symporter |
|  | Population4 | *rph* | Ile225fs | ribonuclease |
|  | Population4 | *glnG* | Val18Leu | transcriptional dual regulator expressed under nitrogen limitation |
| Magnesium-limitation  Environment^€^ | Population1 | *insH* | His7fs | transposase |
|  | Population1 | *yhaV** | Synonymous change:114A>G | YhaV toxic ribonuclease |
|  | Population 2 | *wecF* | Leu90fs | ECA polysaccharide chain elongation |
|  | Population 2 | *ybgK* | Met9Arg | predicted enzyme subunit |
|  | Population 2 | *lptG* | Leu329Arg | lipopolysaccharide transport system |
|  | Population 2 | *phoQ* | Leu467Pro | bifunctional sensory histidine kinase |
|  | Population3 | *nmpC* | Phe345fs | outer membrane porin protein |
|  | Population3 | *hdfR* | Pro251Leu | HdfR DNA-binding transcriptional dual regulator |
|  | Population3 | *yhaV* | Asn39fs | YhaV toxic ribonuclease |
|  | Population3 | *ydbA* | Thr839His | predicted outer membrane protein |
| Low Nitrogen Magnesium Limitation Environment  (LNML) | Population 1 | *rho* | Gly63Val | transcription termination factor |
|  | Population 2 | *lptB* | His195Gln | lipopolysaccharide transport system |
|  | Population 2 | *frlB* | Met163Leu | fructoselysine 6-phosphate deglycase |
|  | Population 2 | *yiiG* | Ile90fs | conserved protein |
|  | Population 2 | *bluF* | Ile304Thr | blue light-responsive regulator of BluR |
|  | Population 2 | *mcrB* | Ser120fs | MrcB subunit of 5-methylcytosine restriction system |
|  | Population 2 | *yggT* | Leu21fs | predicted inner membrane protein |
|  | Population 2 | *gfcD* | Thr330Arg | putative lipoprotein |
|  | Population 2 | *sppA* | Gly514Cys | protease IV, a signal peptide peptidase |
|  | Population 2 | *fimE* | Val169fs | regulator for fimA |
|  | Population 2 | *gcvR* | Asn23His | GcvR predicted transcriptional regulator |
|  | Population 2 | *nlpC* | Cys62fs | lipoprotein hydrolase |
|  | Population 2 | *argI* | Lys34Gln | ornithine carbamoyltransferase chain I |
|  | Population 2 | *lptA* | .*186Ser | lipopolysaccharide transport system |
|  | Population 2 | *xylA* | Ile398Ser | xylose isomerase |
|  | Population 2 | *yhaV* | Lys47Thr | YhaV toxic ribonuclease |
|  | Population 2 | *cysP* | Ser32Tyr | periplasmic binding protein CysP |
|  | Population 2 | *adeD* | Phe86Ser | cryptic adenine deaminase |
|  | Population3 | *gadA* | Gly215Cys | glutamate decarboxylase A |
|  | Population3 | *tolA* | Val411fs | inner membrane protein |
|  | Population3 | *mgtA* | Val501Phe | Mg^2+^ / Ni^2+^ transporting ATPase |
|  | Population3 | *lptA* | Ile36Asn | lipopolysaccharide transport system |
|  | Population3 | *adeD* | Val479fs | cryptic adenine deaminase |
|  | Population4 | *shiA* | Leu260fs | shikimate:H^+^ symporter |
|  | Population4 | *melB* | Ser403fs | melibiose:H^+^/Na^+^/Li^+^ symporter |
|  | Population4 | *sohB* | Tyr51fs | predicted inner membrane peptidase |

** Synonymous mutation is mentioned for this gene because mutations in this gene are repetitive for different populations evolving under magnesium limiting and LNML environment*

^€^ *Population 4 that evolved under magnesium-limiting conditions had low sequence coverage and hence was not included in the analysis.*
